# A Low-Background, High-Flatness Mounting Method for In Situ SIMS Isotopic Analysis for Fine-Grained Samples

**DOI:** 10.1021/acs.analchem.5c04185

**Published:** 2025-09-15

**Authors:** Yu-Bing Gao, Jia-Long Hao, Zhan Zhou, Hui-Cun He, Guo-Qiang Tang, Sen Hu, Wei Yang, Yang-Ting Lin

**Affiliations:** † Key Laboratory of Earth and Planetary Physics, 66451 Institute of Geology and Geophysics, Chinese Academy of Sciences, Beijing 100029, China; ‡ College of Earth and Planetary Sciences, University of Chinese Academy of Sciences, Beijing 100049, China; § State Key Laboratory of Lithospheric and Environmental Coevolution, Institute of Geology and Geophysics, Chinese Academy of Sciences, Beijing 100029, China

## Abstract

Accurate in situ determination of water content and hydrogen isotopes in minerals is essential for understanding magmatic processes on Earth and other planetary bodies. However, such measurements are technically challenging due to low hydrogen concentrations and contamination from atmospheric water vapor. Common mounting methods—such as alloy mounts, indium mounts, and thin sections—are often unsuitable for fine, loose-grained samples. Here, we present a simple and effective mount preparation technique using quartz glass substrates with minimal epoxy resin. This method is optimized for NanoSIMS analysis, achieving ultralow hydrogen background (∼8 ppm) and high-vacuum conditions (∼1.4 × 10^–10^ mbar) with the aid of a custom-designed liquid nitrogen cold trap, while maintaining excellent sample surface flatness. The mounts also proved compatible with high-precision oxygen isotope analysis by large-geometry secondary ion mass spectrometry (LG-SIMS), yielding δ^18^O reproducibility better than 0.30‰ (2SD). This preparation approach enables reliable, high-precision isotopic analysis of fine-grained and extraterrestrial materials and is broadly applicable in microanalytical research.

## Graphical abstract

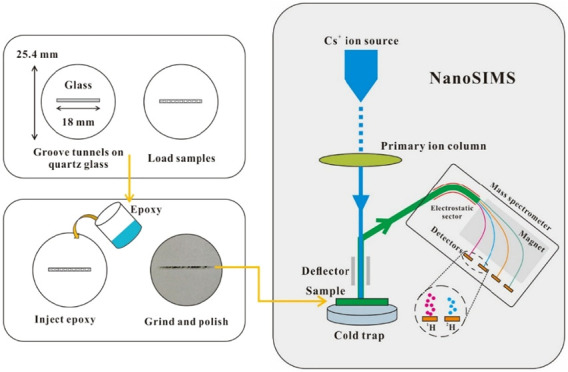

## Introduction

The water content and hydrogen isotopic composition of geological samples are key indicators of magmatic evolution and volatile cycling within planetary bodies.^1–4^ In addition to terrestrial studies, water and hydrogen isotopes have been extensively investigated in extraterrestrial materials from the Moon, Mars, and asteroids.^1,5–10^ In situ measurement techniques including large-geometry secondary ion mass spectrometry (LG-SIMS) and NanoSIMS enable direct quantification of water content in minerals. Compared to LG-SIMS, NanoSIMS offers superior spatial resolution, making it particularly suitable for measuring fine-grained samples. Furthermore, its multicollection capability provides enhanced isotopic discrimination, especially for low-mass elements (e.g., H, C, N, O). These technical advantages have established NanoSIMS as the dominant technique for water content measurement in geological materials. NanoSIMS requires ultrahigh vacuum conditions to minimize contamination from atmospheric water vapor during analysis. Maintaining such vacuum conditions during in situ analysis places stringent demands on sample mounting methods.

Currently, four main mounting strategies are commonly used: epoxy resin mounts, indium mounts, Sn–Bi alloy mounts, and thin sections.^11–14^ The high outgassing rate of epoxy resin can cause a significant increase in hydrogen background levels during NanoSIMS analysis. Indium mounts are suitable for large samples but require prepolishing and are incompatible with particles smaller than ∼1 mm. Sn–Bi alloys eliminate the need for prepolishing and can accommodate various sample sizes, but their poor fluidity and low hardness often lead to incomplete encapsulation and surface protrusion of small particles. Thin sections allow mounting of small grains with minimal epoxy, but the process is technically demanding and samples are prone to detachment during final polishing.

To address these limitations, we introduce a new mounting method specifically designed for small and loosely aggregated particles, such as CE6 lunar soil. This technique employs quartz glass substrates and a minimal amount of epoxy resin to ensure both high vacuum compatibility and mechanical stability. Although developed for NanoSIMS applications, the method is equally applicable to other LG-SIMS that require low hydrogen backgrounds and high surface flatness. In this study, we further quantify the allowable epoxy resin volume that maintains ultralow hydrogen backgrounds, providing a robust preparation protocol for a range of high-precision in situ isotopic analyses.

## Experimental Section

### Samples

Five mineral and glass standards were used in this study: Kovdor apatite (H_2_O = 0.98 ± 0.07 wt %, δ*D* = −66 ± 21‰), Durango apatite (H_2_O = 0.0478 wt %, δ*D* = −120 ± 21‰), OA-1 glass (H_2_O = 1.2 wt %), MORB glass (H_2_O = 0.258 wt %, δ*D* = −73 ± 2‰), and San Carlos olivine (H_2_O ≈ 1 ppm).^12,15–18^ These standards were used to construct the water calibration curve, evaluate hydrogen isotope fractionation, and assess background hydrogen levels. San Carlos olivine also served to test oxygen isotope measurement stability and mount flatness. Hydrogen isotopic compositions in this study are expressed using delta notation (δ*D*), defined as

1 $$\delta D\;\left(‰\right)=\left[{\left(D/H\right)}_{\mathrm{measured}}/{\left(D/H\right)}_{\mathrm{SMOW}}-1\right]\times 1000$$

where (*D*/*H*)_SMOW_ = 1.5576 × 10^–4^ represents the standard hydrogen isotope ratio of Standard Mean Ocean Water (SMOW).

### Mount Preparation

Mount preparation involved embedding fine-grained mineral particles into quartz glass slides using minimal epoxy to ensure low background and high surface flatness. Quartz slides (1 in. diameter, heat-resistant, sourced from Taobao) were paired with EpoxiCure 2 epoxy resin (BUEHLER). Trenches (∼1.8 mm long, 400 μm wide, 400 μm deep) were milled into the quartz surface using a CNC dicing saw (SYJ-400, Shenyang Kejing) with a 100 mm × 12.7 mm × 0.33 mm blade.

To evaluate the effect of epoxy volume on hydrogen background, mounts with 1, 2, 3, and 5 trenches (W1 to W5) were prepared, corresponding to estimated epoxy volumes of ∼0.3, 0.6, 0.9, and 1.5 mm^3^, respectively. An epoxy-free Sn–Bi alloy mount (IGG 104) served as a baseline control.

Standard grains were loaded into each trench and packed with additional olivine or pyroxene particles to minimize voids. A small volume (0.075–0.1 g) of epoxy resin was added, and the mounts were placed in a vacuum oven at 50 °C for 10 min to degas air bubbles. During degassing, buoyant particles were manually repositioned using tweezers. Afterward, the mounts were cured at 50 °C for 6 h.

Following curing, excess epoxy was ground away using a diamond grinding disc to expose the sample surface. The mount surface was then polished with diamond suspension to achieve a smooth finish. Polished mounts were ultrasonically cleaned in ethanol and ultrapure water (2 min each) and dried at 50 °C for 6 h. To prepare for SIMS analysis, a 30 nm gold coating was applied to the surface to optimize both conductivity and experimental efficiency, and the mounts were baked under vacuum for at least 2 days.

This method enables secure embedding of small or loosely aggregated particles while minimizing epoxy use, ensuring compatibility with high-vacuum SIMS analysis and preserving mount integrity during polishing.

### SEM Analysis

Surface topography was evaluated using secondary electron (SE) imaging on a FEI Nova NanoSEM 450 (15 kV, 6.4 nA) at the Institute of Geology and Geophysics, Chinese Academy of Sciences (IGGCAS). This analysis was performed to assess the surface relief between epoxy and mineral grains and to determine whether polishing quality was sufficient for subsequent isotopic measurements. The SE signal, which originates from a shallow surface depth (∼5–10 nm), is highly sensitive to surface topography and therefore provides direct visual confirmation of mount flatness.

### NanoSIMS Analysis

Water and hydrogen isotope measurements were performed using a NanoSIMS 50L at IGGCAS. Mounts were prebaked at 60 °C for 24 h in the airlock and then transferred into the chamber, where they equilibrated for 30 min prior to analysis. A Cs^+^ primary ion beam (500 pA, ∼500 nm spot size) was used in multicollector mode to detect ^1^H^–^, ^2^D^–^, ^12^C^–^, and ^18^O^–^ using four electron multipliers. The energy of the Cs^+^ beam is 8000 V. Surface contamination was removed by presputtering with a 3 nA beam over a 15 × 15 μm^2^ area for 2 min. Data were acquired from the central 3.5 × 3.5 μm^2^ region within a 5 × 5 μm^2^ analytical area (with ∼50% blanking), over 20 cycles of 20 blocks (∼8 min per analysis). An electron gun was used for charge compensation during measurements.

To further reduce the hydrogen background, a custom-designed liquid nitrogen cold trap, developed in-house at IGGCAS, was installed in the analysis chamber to enhance water vapor adsorption and improve the vacuum level.^19,20^ The configuration and performance of the cold trap system are provided in the Supporting Information (Figure S1).

### LG-SIMS Analysis

Oxygen isotope ratios were measured using a LG-SIMS (CAMECA IMS 1280HR) at IGGCAS in Gaussian illumination mode with a Cs^+^ beam (1 nA, <20 μm). Signals of ^18^O^–^ and ^16^O^–^ were collected using dual Faraday cups (10^10^ and 10^12^ Ω amplifiers). The analysis included 60 s presputtering, 60 s centering, and 80 cycles (1.5 s integration). San Carlos olivine on mount W2 was used to assess the influence of surface flatness on δ^18^O values.

## Results and Discussion

### Vacuum Performance and Hydrogen Background

Reliable analysis of low water content samples by NanoSIMS requires minimizing hydrogen background, which in turn demands ultrahigh vacuum conditions. To achieve this, the mounts were baked in a vacuum oven for 2 days, followed by an additional 24 h pumping stage in the airlock chamber. A liquid nitrogen cold trap installed on the analysis chamber further reduces residual water vapor. As a result, the NanoSIMS analysis chamber routinely reached vacuum levels as low as 1.4 × 10^–10^ mbar.

No measurable influence of epoxy resin volume on vacuum stability was observed across mounts with different tunnel numbers (Table S1). To assess hydrogen background more precisely, the ^1^H^–^/^18^O^–^ ratios were measured on San Carlos olivine (SCOl) standards mounted with varying epoxy volumes. As shown in Figure 1, the ratios remained consistent (∼5.88 × 10^–4^) for mounts with one and two tunnels, comparable to the epoxy-free mount (IGG 104). A noticeable increase (to 1.21 × 10^–3^) was observed on the triple-tunnel mount, indicating elevated hydrogen background due to excess epoxy. These results suggest that residual epoxy resin volumes below approximately 0.6 mm^3^ are acceptable for maintaining low hydrogen background during NanoSIMS analysis of hydrous samples. Therefore, the trench depth and width can be flexibly adjusted without predefined dimensional constraints.

**Figure 1:**
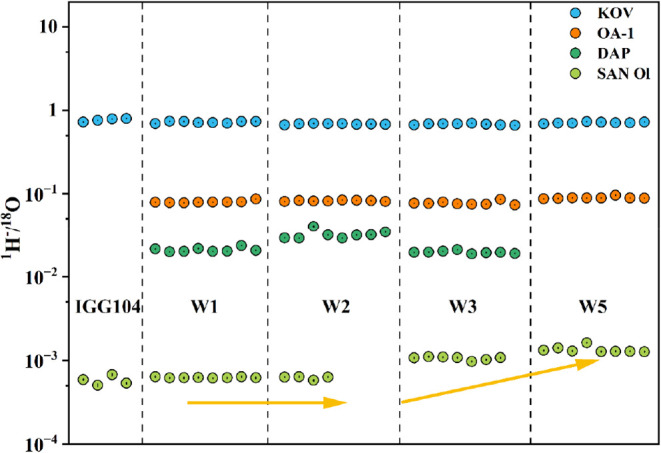
Measured ^1^H^–^/^18^O^–^ values of KOV, OA-1, DAP and SCOl with different amounts of residual epoxy resin. The ^1^H^–^/^18^O^–^ values of the single and double trench mounts and the alley standard mount are consistent. A slight increase is observed on the SCOl of the triple-trench mount. The solid vertical lines represent the standard error (2SE) for each analytical point.

### Calibration of Water Content

To evaluate the impact of residual epoxy resin on water calibration accuracy, NanoSIMS measurements were conducted on KOV, DAP, OA-1, MORB, and SCOl standards mounted with varying epoxy volumes. Water contents were calibrated using the ^1^H^–^/^18^O^–^ signal ratio, following the linear equation

2 [H−1/O−18]=α×[H2O]+β

The ^1^H^–^/^18^O^–^ values were measured directly, while the H_2_O contents were taken from literature. Notably, background hydrogen was not subtracted prior to regression.

As shown in Figure S2, the fitted slope values (α) across different mounts are consistent within analytical uncertainty: 0.743 ± 0.019, 0.702 ± 0.004, 0.702 ± 0.019, 0.726 ± 0.001, and 0.783 ± 0.040 (2SD), with no significant dependence on epoxy volume. The slightly higher α value observed in the epoxy-free IGG 104 mount likely reflects instrument fluctuation during that session. The intercepts (β) are close to zero for all mounts (ranging from −0.008 to +0.001), indicating minimal deviation from linearity.

When the calibration is constrained to pass through the origin (i.e., assuming zero ^1^H^–^/^18^O^–^ at zero H_2_O), the derived slopes remain consistent (0.694–0.778), and background levels can be estimated from the ^1^H^–^/^18^O^–^ ratio measured on San Carlos olivine (SCOl). The corresponding background water contents are8 ppm for W1, W2, and IGG 104; 15 ppm for W3; and 20 ppm for W5.

These results confirm that epoxy resin volumes up to 0.6 mm^3^ do not impact the calibration curve or background water signal. Even with epoxy volumes up to 1.5 mm^3^, no significant distortion of the water calibration curve was observed. The measured background water content of ∼8 ppm agrees well with values reported in previous studies.^12^

### Hydrogen Isotope Fractionation

To assess whether residual epoxy affects instrumental mass fractionation (IMF) of hydrogen isotopes, we measured the *D*/*H* ratios of Kovdor apatite and OA-1 glass. IMF was calculated as

3 $$\mathrm{IMF}=1000\times \left[{\left(D/H\right)}_{\mathrm{measured}}/{\left(D/H\right)}_{\mathrm{standard}}-1\right]$$

The δ*D* values for Kovdor apatite ranged from −147 ± 24 to −119 ± 31‰, while those for OA-1 glass ranged from −287 ± 56 to −222 ± 72‰ (Figure 2). The calculated IMF values for Kovdor apatite varied between 0.9131 ± 0.0282 and 0.9428 ± 0.0354 (2SD). Although OA-1 glass lacks an absolute hydrogen isotope calibration, previous studies have shown that glass and apatite generally exhibit comparable IMF behavior.

**Figure 2:**
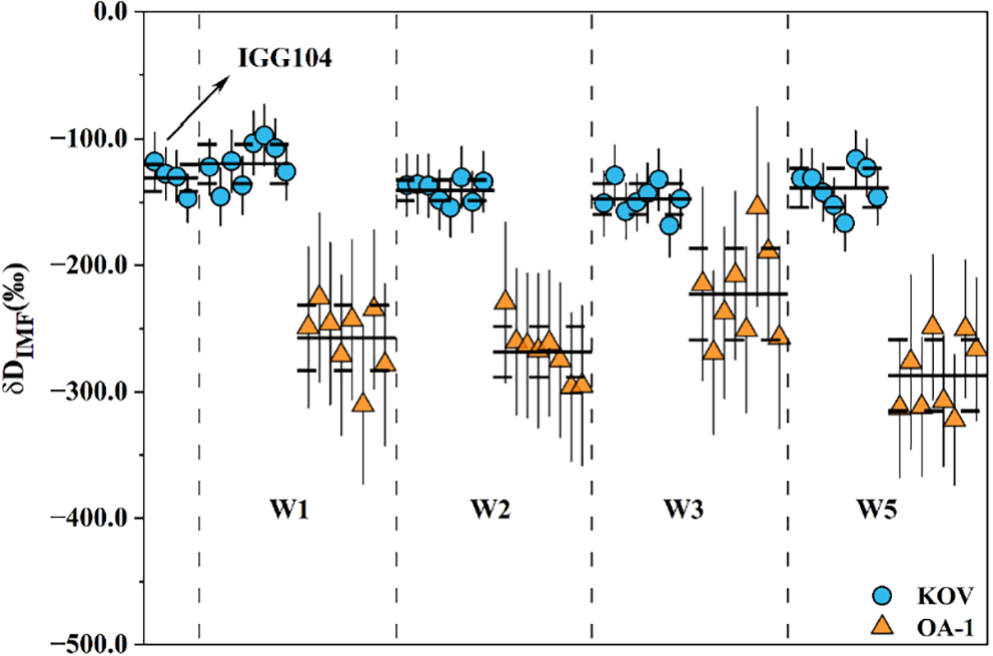
Instrumental mass fractionation (IMF) of the hydrogen isotope on standard samples KOV and OA-1 with different residual epoxy resin. The solid horizontal lines represent the average value, and the horizontal dashed lines represent the standard deviation (2SD) for each group data. The solid vertical lines represent the standard error (2SE) for each analytical point.

The precision for Kovdor apatite measurements was better than 39‰ (2SD), consistent with earlier reports.^12^ These results confirm that up to 1.5 mm^3^ of residual epoxy has no discernible effect on the accuracy or precision of hydrogen isotope measurements.

### Mount Surface Flatness and Its Influence on Isotopic Precision

Geological particles often protrude above the mounting surface after polishing due to differences in material hardness. The trench-mount method effectively mitigates this issue. Secondary electron (SE) imaging using a Nova SEM and microscope images (Figure 3) shows that particles embedded in trench mounts are flush with the epoxy surface, whereas particles in conventional epoxy mounts protrude noticeably.

**Figure 3:**
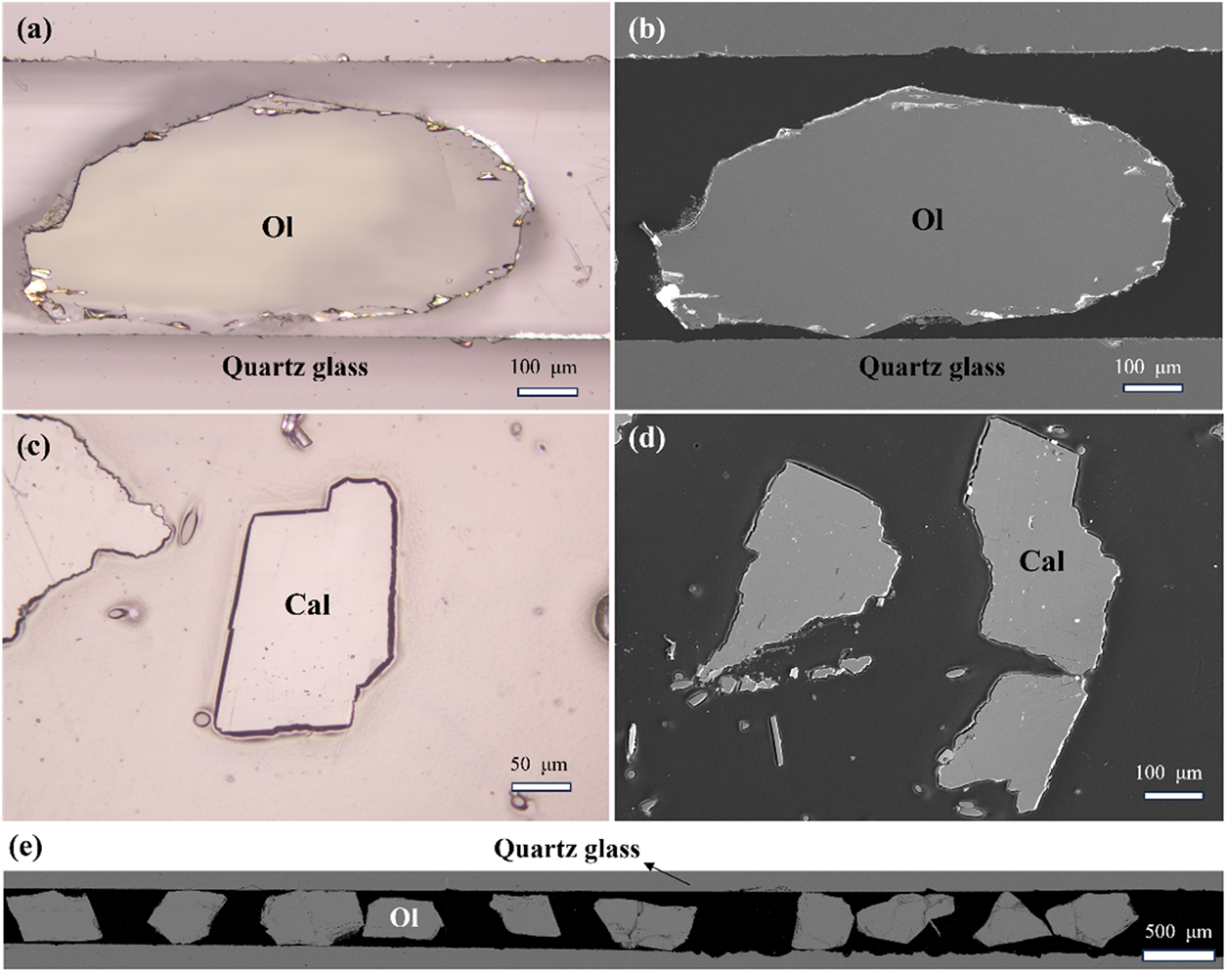
(a) Microscope image of olivine in W3 mount. (b) The SE image of olivine in W3 mount. (c) The microscope image of calcite in epoxy mount. (d) The SE image of calcite in epoxy mount. (e) The BSE image of olivine in a trench.

Surface flatness is critical for high-precision isotopic analyses, particularly for oxygen isotopes. To evaluate this, we performed δ^18^O measurements on San Carlos olivine (SCOl) across the trench mount (W2) using SIMS 1280 (Figures 4 and S3).^21^ The average δ^18^O was 11.73 ± 0.30‰, with an internal precision better than 0.30‰ (2SD). No spatial variation was observed across the mount, confirming that the new preparation method yields flat, uniform surfaces suitable for precise isotopic analysis.

**Figure 4:**
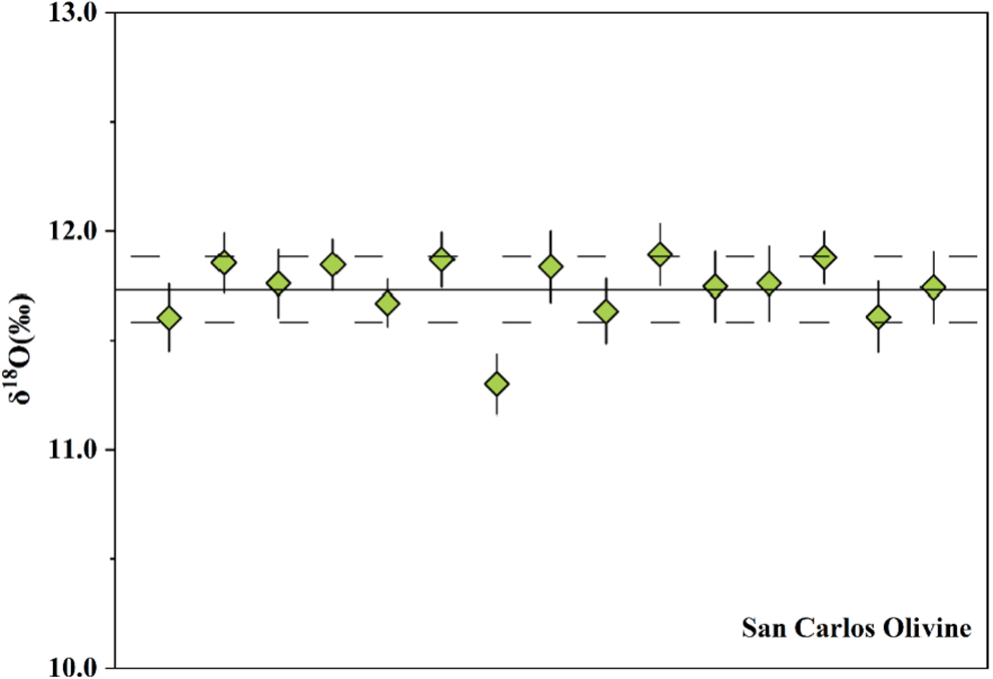
δ^18^O value of standard sample SCOl at different positions in the W2 mount. The solid horizontal lines represent the average value, and the horizontal dashed lines represent the standard deviation (2SD). The solid vertical lines represent the standard error (2SE) of analysis.

## Conclusions

We presented a novel mount preparation method for loose and small particles. The SE images and the stability of δ^18^O measurement results indicate that the mount surface is highly flat. Compared with alley mounts, indium mounts, and the thin-sections, this sample mounting method is easier to prepare and does not require prepolishing. Measurement results show that epoxy resin volumes less than 0.6 mm^3^ have no significant effect on the H background of the NanoSIMS instrument, while volumes less than 1.5 mm^3^ have no significant effect on the analysis vacuum level, H isotope fractionation, or the water calibration curves. Therefore, this is an ideal method for sample mounting method of loose and small particles for water analysis using NanoSIMS. It is particularly well suited to preparing small extraterrestrial particles, such as CE6 lunar soil samples. This protocol is applicable not only to H analysis but also to other volatile elements requiring ultralow background conditions, including C, N and Cl.

## Supplementary Material



## Notes

Additional experimental details for sample preparation; configuration of LN_2_ cold-trap system; calibration curve for water analysis under different epoxy volumes; SIMS oxygen isotope measurement points; vacuum data under different mounting conditions; BSE/SE images of mounted samples (PDF)
