# Supplementary material for: A Low-Background, High-Flatness Mounting Method for In Situ SIMS Isotopic Analysis for Fine-Grained Samples
Source: Anal Chem. 2025 Sep 15;97(39):21200–4. doi: 10.1021/acs.analchem.5c04185 (PMC12509191; doi:10.1021/acs.analchem.5c04185)
Supplement: Supplementary file 1 [file ac5c04185_si_001.pdf]

Supporting Information for

# A Low-Background, High-Flatness Mounting Method for In Situ SIMS Isotopic Analysis for Fine-Grained Samples

Yu-Bing Gao [a, b], Jia-Long Hao [a*], Zhan Zhou [a, b], Hui-Cun He [a], Guo-Qiang Tang [c], Sen Hu [a], Wei Yang [a] and Yang-Ting Lin [a]

[a] Key Laboratory of Earth and Planetary Physics, Institute of Geology and Geophysics, Chinese Academy of Sciences, Beijing, 100029, China

[b] College of Earth and Planetary Sciences, University of Chinese Academy of Sciences, Beijing 100049, China

[c] State Key Laboratory of Lithospheric and Environmental Coevolution, Institute of Geology and Geophysics, Chinese Academy of Sciences, Beijing 100029, China

* Email: sean_hao@mail.iggcas.ac.cn (*J-L. Hao)

**Table of Content**

Additional experimental details for sample preparation; configuration of $LN_2$ cold-trap system; calibration curve for water analysis under different epoxy volumes; SIMS oxygen isotope measurement points; vacuum data under different mounting conditions.

**Figure S1.** Configuration and vacuum performance of the custom-designed cold trap system

**Figure S2.** Water calibration curve with different residual epoxy resin

**Figure S3.** $\delta^{18}O$ measurement points on SCOl

**Table S1.** Measurements of analysis chamber vacuum of NanoSIMS and $^{1}H^{-}/^{18}O^{-}$ on SCOl under different residual epoxy resin

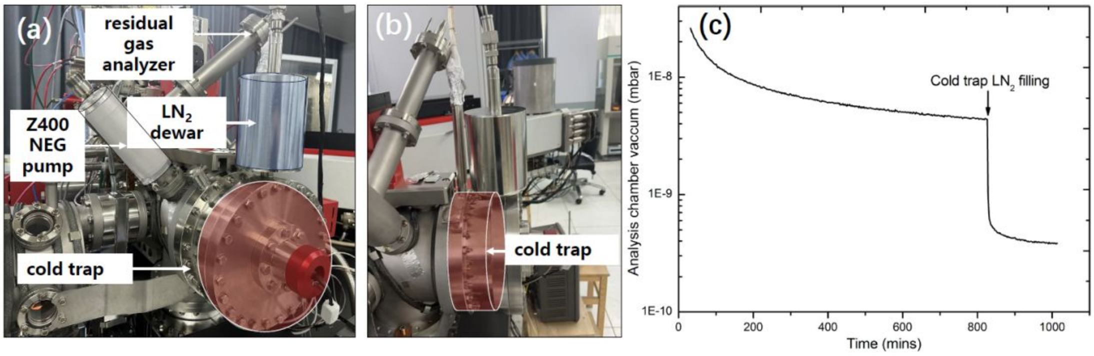


**Figure S1.** Configuration and performance of the custom $LN_2$ cold-trap system. (a) Photograph of the NanoSIMS chamber showing the custom cold trap, $LN_2$ Dewar, Zr-based non-evaporable getter pump (Z400), and residual gas analyzer (RGA).

(b) Side view of the straight-line $LN_2$ cold trap mounted on an extended flange of the analysis chamber. The trap incorporates an annular internal structure, allowing the primary ion beam to pass through unobstructed and reach the FCo primary beam detection system, while efficiently capturing residual $H_2O$ vapor.

(c) Chamber pressure as a function of time measured using a resin-mounted test sample. After $LN_2$ filling of the cold trap (arrow), the pressure rapidly improves from ~4–5 × $10^{-9}$ mbar to ~3–4 × $10^{-10}$ mbar, demonstrating the effectiveness of the cold trap. Under optimized quartz-substrate mounts, the analysis chamber routinely reaches ~1.4 × $10^{-10}$ mbar.

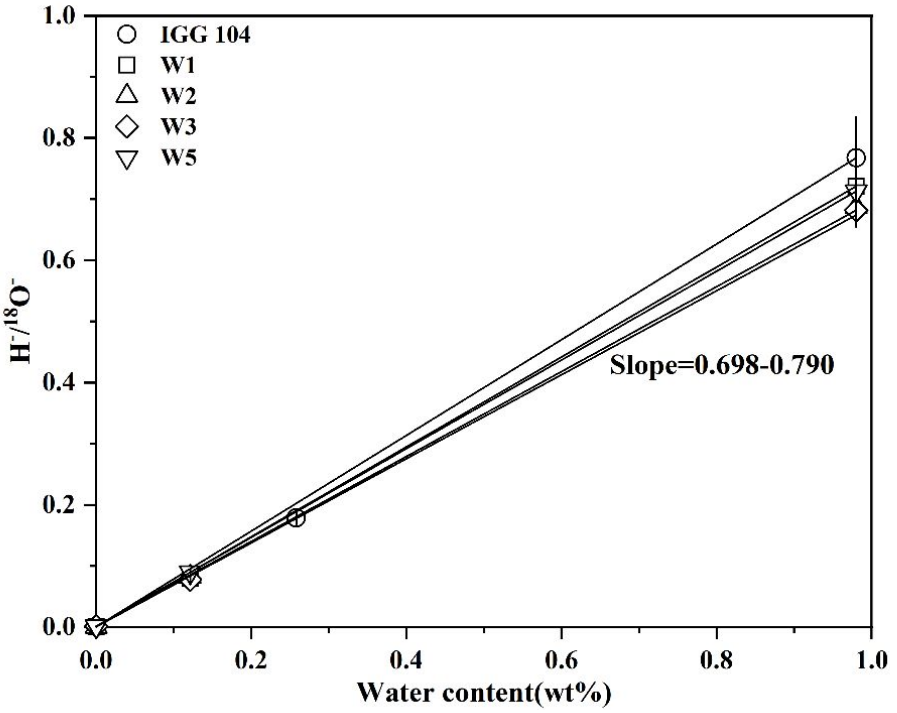


**Figure S2.** Water calibration curve with different residual epoxy resin.

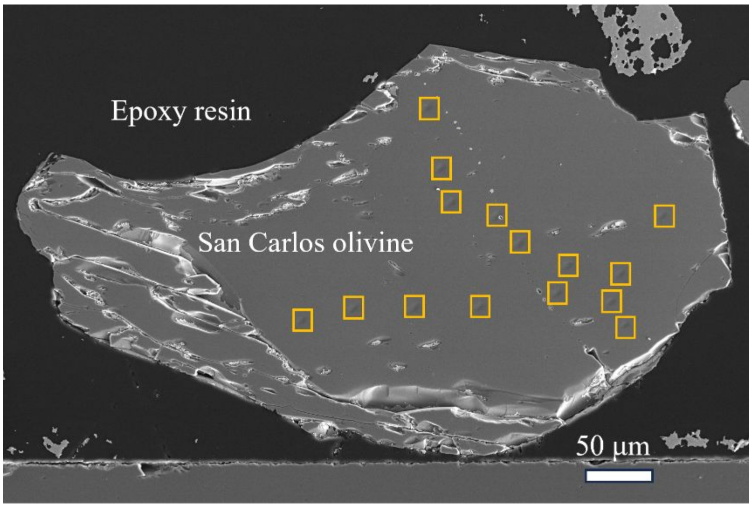


**Figure S3.** The SE image of SCOl in W2 mount. The orange rectangles are the measurement points for SIMS oxygen isotope analysis.

**Table S1.** Measurements of analysis chamber vacuum of NanoSIMS and $^{1}H^{-}/^{18}O^{-}$ on SCOl under different residual epoxy resin.

| | **Mount#** | **Residual epoxy (Vol. mm$^3$)** | **Vaccum (mbar)** | **Backgroud ($^{1}H/^{18}O$)** | **2 SD** |
|---|---|---|---|---|---|
| Epoxy resin | W1 | ~0.3 | $1.4\times10^{-10}$ | $6.24\times10^{-4}$ | $1.74\times10^{-5}$ |
| | W2 | ~0.6 | $1.4\times10^{-10}$ | $5.66\times10^{-4}$ | $2.39\times10^{-4}$ |
| | W3 | ~0.9 | $1.4\times10^{-10}$ | $1.21\times10^{-3}$ | $8.74\times10^{-4}$ |
| | W5 | ~1.5 | $1.4\times10^{-10}$ | $1.34\times10^{-3}$ | $2.45\times10^{-4}$ |
| Sn-Bi alloy | IGG 104 | 0 | $1.4\times10^{-10}$ | $5.75\times10^{-4}$ | $1.50\times10^{-4}$ |
